# InN Nanowires: Growth and Optoelectronic Properties

**DOI:** 10.3390/ma5112137

**Published:** 2012-10-31

**Authors:** Raffaella Calarco

**Affiliations:** Paul-Drude-Institut für Festkörperelektronik, Hausvogteiplatz 5-7, 10117 Berlin, Germany; E-Mail: calarco@pdi-berlin.de; Tel.: +49-30-20377-351; Fax: +49-30-20377-201.

**Keywords:** self-assembly semiconducting, molecular beam epitaxy (MBE), nanoscale, electrical properties, III-V, optical properties, optoelectronic, photoconductivity

## Abstract

An overview on InN nanowires, fabricated using either a catalyst-free molecular beam epitaxy method or a catalyst assisted chemical vapor deposition process, is provided. Differences and similarities of the nanowires prepared using the two techniques are presented. The present understanding of the growth and of the basic optical and transport properties is discussed.

## 1. Introduction

Nanowires (NWs), as the word itself suggests, are objects that are extended in one dimension (up to several micrometers) and have a cross-section in the nanometer range. The very first demonstration of semiconductor nanowire growth using the vapor-liquid-solid catalytic (VLS) method can be traced back to 1964 [1], but not until the 90s did nanowires activate a large interest in a broad community ranging from physicists and chemists to material scientists and engineers.

The scientific beauty and fascination of nanowires lie in their single crystalline nanostructure and the possibility of using simple and low-cost growth methods. An additional advantage of NW heteroepitaxy, for instance, is that a broad variety of material combinations is possible because in nanowire synthesis the formation of dislocations originating from lattice mismatch could eventually be prevented. Thus, due to elastic relaxation of NWs greater lattice mismatch could be accommodated through pseudomorphic growth without defect introduction when compared to traditional two-dimensional thin film growth, opening new routes for the integration of optoelectronics on silicon. In addition, nanowires as well as nanowire heterostructures can be fabricated on a wide variety of substrates, including silicon, which make them to some extent suitable for future Complementary Metal Oxide Semiconductor integration. For a detailed overview on nanowires please see also references [2,3,4,5].

Furthermore, due to their quasi one-dimensional structure, nanowires exhibit interesting electronic properties. Although sophisticated device structures have already been realized [6,7,8,9,10,11,12], many fundamental questions remain unexplored.

Nitrides are materials very well suited to a variety of optoelectronic applications as they possess a direct band gap. The band gaps of III-nitride alloys cover an extended wavelength range from the near infrared into the visible and up to the ultra-violet. Additionally, outstanding properties of this material system are “polarization doping” and piezoelectricity. It is possible to obtain two-dimensional carrier densities of about 10^13^ cm^−2^ without doping, only due to a discontinuity of the internal polarization at the heterojunction. Thus, electron velocities larger than 2 × 10^7^ cm/s have been reached [13]. Nitride transistors have indeed already shown excellent performance in planar structures. Adding up these entire qualities one can conclude that nitrides represent one of the most versatile semiconductor material systems. However, due to the lack of homoepitaxial substrates for nitride epitaxy the crystalline quality of planar films is not perfect. Despite high defect densities nitride devices are extraordinarily performing. NWs, which can be grown as single nano-crystals, show fewer structural defects than planar films. Therefore, NWs are expected to further improve the device quality. In summary, nitride NWs represent an attractive playground for investigating interesting physical issues ranging from fundamental research up to applications and subjects more relevant for industry.

In this review particular emphasis is given to the presentation of effects due to surface space charge layers, especially focusing on InN NWs grown by molecular beam epitaxy (MBE). GaN NWs have already been extensively covered by several reviews [14,15,16,17].

InN was the last studied material of the III-nitride alloy family. Firstly, this is due to the limited crystallographic and optical quality of the grown InN films, and secondly to the fact that its band gap value was supposed to be around 1.89 eV as other established materials, such as the group III-arsenides and -phosphides. The determination of the former band gap value was provided on the basis of absorption measurements [18]. Later, after an improvement in film quality [19], strong luminescent InN films grown by MBE were reported [20], and the value of the band gap was below 1 eV. In addition, the value of the electron concentration decreased from typically 10^20^ cm^−3^ to 10^18^ cm^−3^ with mobilities of the order of 1000 cm^2^/Vs at room temperature [21]. Furthermore, the value of other fundamental properties like the electron effective mass was revised from 0.11m_0_ to 0.07m_0_. The experimental results pointing at a low fundamental band gap of InN (<0.7 eV) was supported by theoretical calculations by Bechstedt *et al*. [22]. Furthermore, Wu *et al*. showed the absorption edge of InN is affected by the Moss-Burstein shift. Actually the absorption edge shifts to 1.7 eV in case of a free electron concentration of 4.5 × 10^20^ cm^−3^ [21,23]. The high electron concentration causes a band-filling of the conduction band and since absorption of a photon only occurs if an electron is excited from an occupied to an unoccupied state, the required photon energy increases with the shift of the Fermi level.

## 2. Results and Discussion

InN NWs crystallize in the wurtzite structure. Two possible orientations have been achieved. In the first orientation, the NW axis is oriented perpendicular to the c-plane (polar), and the sidewalls are formed by M-plane facets (non-polar). Such NWs are usually fabricated by means of molecular beam epitaxy (MBE) [24]. In the second orientation, the NW axis is oriented along the [11-20] direction and therefore the sidewalls are formed by the polar {0001} and semipolar {1-101} facets [25]. It is worth mentioning here that those NWs are VLS fabricated using a furnace or chemical vapor deposition (CVD).

### 2.1. Fermi Level Pinning and Internal Electric Field

**Figure 1:**
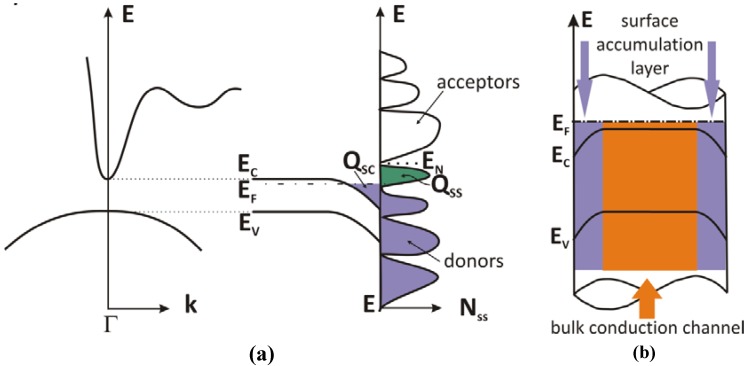
(**a**) Band structure of a narrow gap semiconductor depicted qualitatively together with the corresponding surface density of states. The charge accumulation layer (depicted in violet) is shown; (**b**) Schematic representation of the band diagram for InN nanowires (NWs). The violet areas in the wire correspond to the surface accumulation layer, which extends only a few nm. The orange area corresponds to the bulk conduction channel. The relative positions of E_F_, E_C_, and E_V_ are not to scale.

At the surface of a 3D crystal the periodicity of its structure is broken, and the electronic potential has to reach the vacuum level. This change in potential induces a different behavior of electrons at the surface with respect to that in the bulk, which is described by surface electronic states. The surface states can carry charge, the sign of which depends on the type of surface states (acceptor or donor) and on the position of the Fermi level E_F_. Surface states derived from the conduction band E_C_ (acceptor-type) are neutral if empty and negative if occupied. In contrast, surface states derived from the valence band E_V_ (donor-type) are neutral if occupied with an electron and positive if empty. Thus, there must exist a so-called neutrality level E_N_, where the acceptor type of surface states switches over into a donor type (lower half of the forbidden band). When E_F_ coincides with the neutrality level E_N_, the surface states in total are neutral. If the density of surface states (N_SS_) is very high (> 10^12^ cm^−2^), the position of E_F_ at the surface is determined by E_N_ of the surface states and E_F_ is pinned near E_N_. The pinning of the Fermi level is accompanied by the accumulation of charge in the surface states. The condition of charge neutrality at the surface requires that the surface state charge (Q_SS_) is compensated by an opposite charge inside the semiconductor called space charge (Q_SC_), which can be described by the bending of the electronic bands. The bending depends on the surface state charge and in general one can discriminate between two different cases: Depletion, where in an n-type semiconductor the density of free electrons decreases whereas the density of holes (negligible) increases due to the upwards band bending; Accumulation, where in an n-type semiconductor the free electron concentration at the surface is larger than the bulk value due to the downward band bending. This is the case for an n-type narrow band gap semiconductor such as InN, InAs, and InSb; an example of the bending is given in Figure 1a.

The conduction band of a narrow gap semiconductor has a very steep minimum at the Γ point, which accommodates fewer electronic states than the second broad minimum positioned in k-space between the L and M point. The local density of surface states is therefore influenced strongly by the majority of states which is located not at the Γ point, but rather at the side minimum at higher energy. Therefore, the E_N_ lies above the conduction band minimum. For this reason, a positive surface charge Q_SS_ (empty donor states, depicted in green in Figure 1a) is built up. To compensate the positive surface state charge, a negative one has to appear below the surface Q_SC_, therefore free electrons move in the conduction band into the region below the surface. The accumulation of a negative charge at the surface bends the electronic bands downwards as schematically shown in Figure 1a.

The same surface field picture applied to a three dimensional NW structure can be described as a cylinder like surface, marked by an accumulation layer which envelops a bulk conduction channel located in the middle of the wire, as schematically depicted in Figure 1b.

### 2.2. Polarization Fields

Crystals with wurtzite structure exhibit three different types of electrical polarization: Spontaneous polarization, which has non-zero value in absence of an external electric field; induced polarization, which appears if an electric field is applied; direct piezoelectric polarization, developed in presence of stress. Hence, in the absence of external electric fields, the total macroscopic polarization is the sum of the spontaneous polarization in the equilibrium lattice and the strain-induced or piezoelectric polarization.

The spontaneous polarization of the group-III nitrides was calculated by Bernardini *et al*. [26] and it is found to be negative. The sign of the spontaneous polarization is determined by the polarity and turns out to be opposite to the [0001] direction. Piezoelectric constants of GaN, AlN, and InN crystals have been also calculated by Bernardini *et al*. [26]. The calculated values for InN GaN and AlN are shown in Table 1, these are up to ten times larger than in GaAs.

**Table 1:** Spontaneous polarization and piezoelectric constant values for III-nitrides, taken from [26].

III-nitrides	α-InN	α-GaN	α-AlN
Spontaneous polarization (C/m^2^)	−0.032	−0.029	−0.081
Piezoelectric constant e_31_ (C/m^2^)	−0.57	−0.49	−0.60
Piezoelectric constant e_33_ (C/m^2^)	+0.97	+0.73	+1.46

### 2.3. Synthesis of InN NWs

#### 2.3.1. MBE

It is well known that the morphology of MBE-grown-InN thin films depends critically on the III-V flux ratio [27]. In-rich conditions favor the realization of smooth compact layers whereas N-rich growth leads to the formation of rough layers and even to columnar structures and nanowires. It is however necessary to properly choose the combination of the three parameters substrate temperature (T_subs_), N flux and in flux to control the morphology of the wires [28].

T_subs_ is a very sensitive parameter for InN growth due to its low dissociation temperature. The growth temperature for InN is very close to the decomposition temperature, which means that only a tight window for the growth is available. InN already starts to dissociate at around 450 °C in vacuum [29] and decomposes at 630 °C [30] (please note that these values are polarity dependent).

Furthermore, the dissociation temperature of InN can be increased by increasing the pressure of nitrogen in the annealing atmosphere. This means that for InN the nitrogen desorption controls the growth rate, whereas gallium desorption plays a significant role for GaN growth. After InN dissociation, the nitrogen quickly evaporates whereas In atoms are left on the substrate surface. In conclusion, the growth temperature for InN should be carefully chosen.

**Figure 2:**
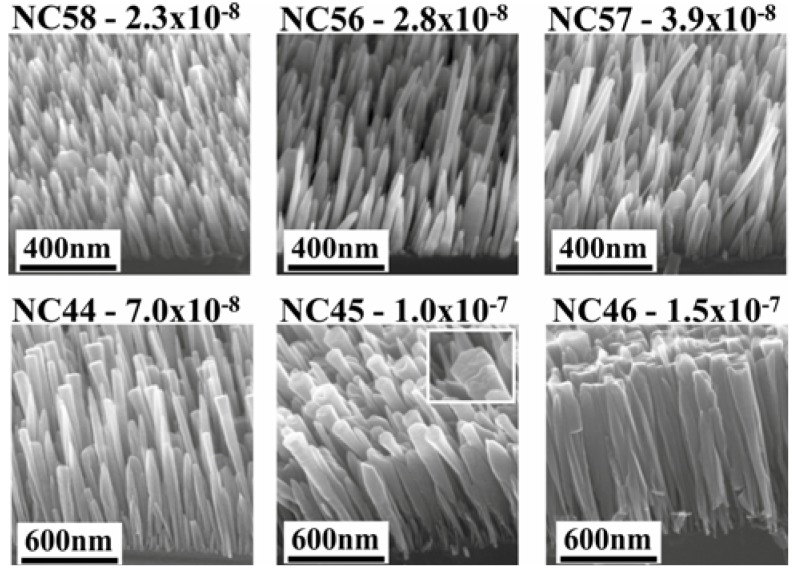
Morphology of molecular beam epitaxy (MBE) grown InN NWs on Si(111) at 475 °C at different in-fluxes. With permission from [24].

Analyzing the morphology of nanowire samples grown at different In fluxes [24] it is possible to identify a window for the in flux (between 2.3 ×∙10^−8^ mbar and 3.9 ×∙10^−8^ mbar BEP) in which the grown NWs show at the same time two different diameter-length distributions (Figure 2). In those samples, long, thin wires, without tapering, which are particularly interesting for transport measurements, can be found. The size distribution of the latter thin and long NWs would give a correlation between length (L) and diameter (d) similar as for the GaN case (L ≈ 1/d) [31,32]. Few NWs are short in length and broad in diameter, those wires do not fit into the proposed model. This discrepancy can be related to the availability of adatoms during growth. It seems that when the thinner wires start to evolve with time by diffusion and their density increases, the growth of adjacent NWs can be limited by the mass transport towards the thinner NWs and there may be virtually no growth. So far, the role played by the neighboring nanowires has not been deeply investigated for InN NWs.

To further address the role of the diffusion for the growth mechanism of InN NWs in [24] the authors analyzed the temperature dependence of the NW growth rate. Growth is enhanced by the contribution of adatom diffusion along the wire sidewalls to the top of the wire, a process which depends very much on temperature. In fact, the growth rate is a function of substrate temperature for a set of InN NW samples grown with the same nominal In and N flux. This effect can be explained by a diffusion mechanism suggesting a similar growth mechanism for InN as determined for GaN nanowires. In conclusion, for the investigated In and N flux ranges, a higher substrate temperature (<dissociation temperature of InN) improves the columnar morphology of the wires, but the dissociation of InN limits the temperature range.

As previously mentioned, the morphology of the NWs is not completely uniform. Thus, to optimize the growth, their optical quality determined by photoluminescence (PL) spectroscopy was considered [24]. The main PL peak has an energetic position at low temperature in the range of 760–820 meV. It was shown that the PL intensity increases with deposition time and In flux. As the PL intensity depends on the excitation volume one should also keep in mind that with increasing the deposition time the NW length is increasing as well. Also, the increase of the in flux is acting on the density of the NWs.

In general, by varying those parameters it is possible to influence the volume of the investigated material, *i.e.*, length and density of the nanowires. However, comparing the intensity increase as a function of growth parameters to the volume increase, it is possible to rule out the contribution due to a larger volume of the excited material. In conclusion it was possible to state that for increasing deposition time and In flux there is also an increase of the intrinsic quality of the nanowires. The better PL quality of longer NWs can be explained by the fact that due to the high absorption the contribution of the wire base (where stacking faults are present) is reduced. This is an indication that the PL properties vary along the column length. For shorter columns with a strong PL contribution from the bottom part, there is a broad peak with a lower intensity due to non-uniform properties along the columns and a high non-radiative recombination at the base of the columns. Furthermore a higher “intrinsic” doping due to Si segregation can be expected at the bottom part of the columns, similar to the case of GaN grown on Si(111) [33].

As in the case of GaN NWs grown on Si(111) a thin amorphous (presumably Si_x_N_y_) layer of approximately few nm at the interface between nanowires and substrate is formed [34]. To achieve epitaxial growth an AlN buffer layer is necessary. Epitaxial relationship between NWs and substrate/buffer was found to be Si(111)║AlN(0001)║InN(0001) and Si[110]║AlN[11-20]║InN[11-20] [34].

Another approach for the synthesis of InN NWs was followed by Mi and coworkers [35] who used the deposition of a thin In seeding layer prior to NW growth. The authors suggest that in presumably forms nanoscale droplet that are further providing nucleation centers for the growth process. The obtained NWs are non tapered and fairly homogeneous in size. In addition, in this case the growth direction is parallel to the [0001] direction.

InN NWs with fairly large diameters (200 nm) have been also achieved using selective area growth in holes performed via focused ion beam in GaN templates. The technique used for growth is the electron cyclotron resonance plasma-exited MBE [36]. Selective area growth of InN by MBE using a Mo mask was successfully achieved [37] although the NWs display fairly large diameters of the order of the micrometer and dislocation density of 10^9^−10^10^ cm^−2^.

The effect of Si and Mg doping on morphology, electrical and optical properties of InN NWs has been previously reviewed [15].

#### 2.3.2. Vapour-Liquid-Solid Mechanism

Very often for the NW growth, a CVD system [38] or a simple furnace with or without an inert carrier gas is used as experimental equipment [39]. In the last case, the material, in the form of powders, is placed within the furnace. The parameter used to control the growth of the NWs is the oven temperature, which influences the evaporation of the powder.

To fabricate the NWs the vapor-liquid-solid (VLS) mechanism is used, in which a liquid droplet acts as a preferred sink for arriving atoms, and is involved as catalyst in the chemical processes. The liquid droplet becomes supersaturated and the excess of atoms from the vapor is precipitated at the liquid-solid interface. Very often Au colloids, dispersed on a host substrate, are used as collector particles. In this way the NWs grow in a 1D anisotropic fashion. The size of the metal particle determines the initial diameter of the NWs. NW length can also be regulated by the growth time. For a detailed overview on VLS growth mechanism please see, for one example [5].

### 2.4. Optical Measurements

**Figure 3:**
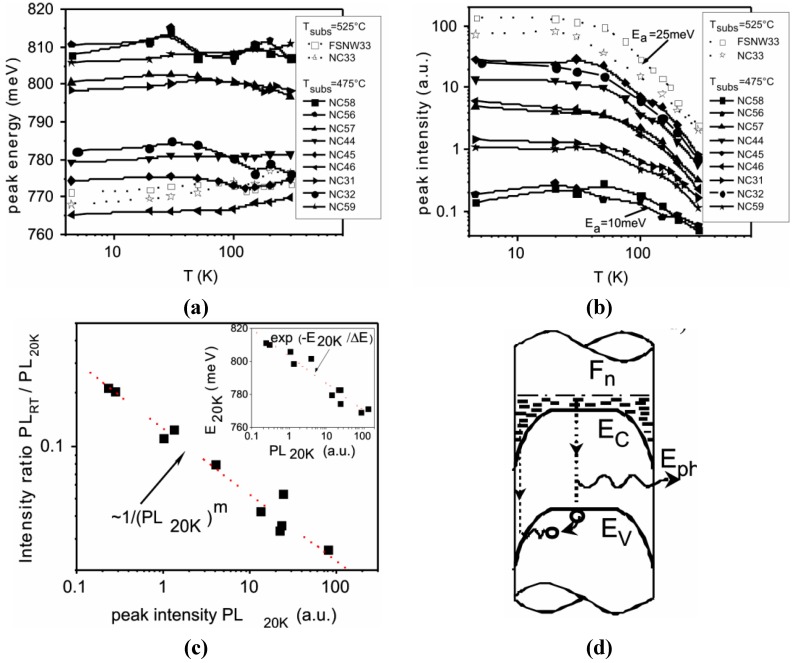
Photoluminescence (PL) Temperature dependence (**a**) peak energy; (**b**) peak intensity; (**c**) RT to 20 K peak intensity ratio *vs.* PL intensity at 20 K. Inset shows the correlation of peak energy to intensity at 20 K; (**d**) schematic band diagram that illustrates the carrier recombination within the accumulation layer of the lateral surface of the wires. With permission from [40].

PL measurements are proven to be useful to optimize the MBE growth process in terms of the optical quality. Furthermore, they allow us to prove the existence of a surface accumulation layer, as expected for a degenerate n-type low bandgap semiconductor. A more detailed analysis of the PL spectra measured for different samples in the temperature range 4–300 K led to the investigation of the basic properties of the InN NWs [40]. A weak influence of the laser intensity on the peak position and the shape of the PL was observed, which can be attributed to a weak band filling effect due to photocarriers. For InN as a degenerate n-type semiconductor, the band filling effect induced by increasing the illumination intensity should be attributed mostly to the minority band of holes. This means that for the conduction band a low photocarrier concentration is given relative to the background doping. The high unintentional doping in InN results in a strong broadening of the band-to-band luminescence spectrum. In addition, the presence of low and high energy tails on the PL peak was detected [40]. Additionally, a thermal quenching of the PL efficiency was observed (Figure 3), which was understood by means of a model based on an accumulation layer at the nanowire surface. Optically excited holes are thermally activated over the potential barrier of the downward bend valance band at the surface and recombine with the high-density electrons of this surface accumulation layer. The low energy tail is in agreement with a distribution of holes in an exponential tail of localized states (Urbach tail) at the edge of the valence band. At low temperatures, the holes are excited from localized states to the valence band upon increasing the temperature. As a result, the PL peak shifts to higher energy. The long tail at high energy of the spectrum at low temperature and the broadening of the PL peak was explained by different phenomena:

1. local potential fluctuations, which cause fluctuations in the band edges;
2. fluctuations of the Fermi level along the wire relative to the conduction band;
3. Landsberg broadening due to the finite time of the final state of the electron-hole recombination.

Of course, in general a superposition of these phenomena should be taken into account. The authors, however, considered only the fluctuation of the Fermi level and focused on modeling the spectral region close to the Fermi level in order to evaluate its position and the related electron concentration. The optical bandgap and the electron concentration of unintentionally doped samples were found in the range 730–750 meV and 8 ×∙10^17^–6 ×∙10^18^ cm^−3^, respectively. Differences between samples were mainly due to variations of the electron concentration (intrinsic doping). As discussed in the previous section a decrease of the background doping level with In flux and growth temperature could be expected and was indeed observed.

This result compares well with those obtained for thin InN layers in which a surface accumulation layer on the (0001) surface has been found [41]. Nevertheless, it should be noted that nanowires consist not only of a (0001) top facet but also of lateral non-polar facets. Theoretical calculations [42] suggest that two-dimensional electron gas (2DEG) would not be expected on InN non-polar surfaces. In the same paper, however, it has been proposed that an In rich surface can lead to a surface 2DEG also for non-polar surfaces. The growth mechanism for MBE samples discussed in the previous section accounts for a strong contribution due to an In adatom diffusion on the nanowires sidewalls; therefore, it is reasonable to assume In rich lateral surfaces.

The light interaction with InN NWs is an interesting and debated topic. The Raman spectrum acquired in a back scattering geometry, from an ensemble of InN NWs in an as grown sample displays in addition to the E_2_^H^, a strong LO peak that appears at a frequency close to E_1_(LO) phonon mode, too low in frequency to be attributed to the L^+^ mode. Some authors [43,44] ascribed it to the symmetry-forbidden E_1_(LO) mode arising from the low-carrier-density region of the nanowire core. Its appearance is explained on the base of a strong scattering of the excitation laser light inside of the nanowire ensemble. In contrast, other interpretations attribute this mode to the accumulation layer [45], or to a particular damping of the plasmonic system [45,46]. Non-polar and unscreened modes are observed in Raman spectra on single NWs almost independently of the scattering geometry [47] ruling out the interpretation given in [43,44] and supporting the interpretation of [45,46]. Furthermore, the absence of the peak in the out of resonance UV spectra [48] supports the latest findings. Recently, a double resonance effect [49] and longer phonon lifetimes [50] in InN NWs have been reported.

### 2.5. Transport

The band diagram for an InN NW with accumulation layer at the lateral facets is depicted in Figure 1b. To prove the presence of a surface accumulation layer, I-V characteristics of InN NW devices were measured and the resistance was displayed as a function of temperature [51]. The curve shows a linear decrease of the resistance with decreasing temperature which is expected for a degenerate semiconductor. This is an indication that the main conductance contribution arises from the electron accumulation layer at the surface. Additional information can be gained by analyzing the slope of the conductance as a function of diameter. A linear dependence is expected for conductance through a surface 2DEG, as the surface increases as π d, while degenerate bulk conductance increases with the cross sectional area of the wire π d^2^. The dependence of the presented data is slightly above linear, therefore suggesting a major conductance contribution through the surface accumulation layer with some additional contribution through the degenerate bulk region, with the Fermi-level located in the conduction band.

The shell conductivity ascribed to an electron accumulation layer at the bare InN surface is investigated more precisely in terms of its chemical composition. It is found that the accumulation layer forms at the interface between InN and In oxide formed during exposure to air [52].

A quantitative determination of surface Fermi level pinning position in MBE InN nanowires is provided using polymer electrolyte gating in combination with a 3D electrostatic modeling of charge distribution [53]. Fermi level 0.6–0.7 eV above the conduction band minimum is found. Doping concentration and carrier mobilities are also evaluated in dependence of the Fermi level pinning [53].

Magnetoconductance oscillations have been measured for MBE InN NWs with diameters below 60 nm [54]. A very pronounced single flux quantum periodicity was found. This kind of periodicity appears to be the same as in an Aharonov-Bohm experiment, in which interference between two separately propagating partial waves takes place. However, the geometry of the contacts applied at the NW ends lead to exclude this interpretation. The magnetoconductance oscillations and the occurrence of the single flux quantum periodicity were attributed to the periodicity of the energy eigenvalues induced by coherent angular momentum states. Based on these investigations, a new type of phasecoherent transport has been clearly demonstrated. The design of future phase-based quantum electronic devices could take advantage of these findings.

A clear semiconductor-to-metal transition at T ~ 80 K is revealed for catalyst-grown InN NWs. Below the transition, the conduction is dominated by 3D Mott variable range hopping [39]. Furthermore, it has been evidenced that the conduction is dominated by a bulk degenerate electron system [55]. The authors also evidenced a size-dependent conduction with a critical side length around 80 nm.

Oxygen sensitization of InN catalyst-grown NWs suggested that oxygen and acceptor defects lead to compensation of the surface shell high electron concentration and bulk core, respectively [25].

## 3. Conclusions

InN NWs fabricated using a catalyst-free molecular beam epitaxy method or a catalyst-assisted CVD process revealed a high crystalline quality. The differences between the crystallographic orientation of the NWs produced using each method were stressed. In case of MBE grown InN NWs, the growth parameters were optimized with respect to the morphology and to the optical properties.

Beyond the presented results it should be mentioned that a lot of specific growth studies revealed many additional details. The deep knowledge acquired in this field thus allows us to confidently address the challenge of growing very complex heterostructures for future nanodevices.

Furthermore, the understanding of basic transport properties was discussed. In particular, the effect of surface Fermi-level pinning and its interplay with the NW dimensions were considered.
